# Methylmercury Risk Assessment Based on European Human Biomonitoring Data

**DOI:** 10.3390/toxics10080427

**Published:** 2022-07-28

**Authors:** Noelia Domínguez-Morueco, Susana Pedraza-Díaz, María del Carmen González-Caballero, Marta Esteban-López, Mercedes de Alba-González, Andromachi Katsonouri, Tiina Santonen, Ana Cañas-Portilla, Argelia Castaño

**Affiliations:** 1 National Centre for Environmental Health, Instituto de Salud Carlos III, 28220 Madrid, Spain; ndominguez@isciii.es (N.D.-M.); mcgonzalez@isciii.es (M.d.C.G.-C.); m.esteban@isciii.es (M.E.-L.); malba@isciii.es (M.d.A.-G.); castano@isciii.es (A.C.); 2 Cyprus State General Laboratory, Ministry of Health, P.O. Box 28648, Nicosia 2081, Cyprus; akatsonouri@sgl.moh.gov.cy; 3 Finnish Institute of Occupational Health, P.O. Box 40, 00032 Työterveyslaitos, Finland; tiina.santonen@ttl.fi

**Keywords:** HBM4EU, human biomonitoring, mercury, methylmercury, risk assessment

## Abstract

A risk assessment (RA) was conducted to estimate the risk associated with methylmercury (MeHg) exposure of vulnerable European populations, using Human Biomonitoring (HBM) data. This RA was performed integrating published data from European HBM surveys and earlier EFSA approaches (EFSA 2012). Children/adolescents (3 to 17 years old) and women of childbearing age (18 to 50 years old) were selected as relevant study population groups for this RA. Two types of HBM datasets were selected: HBM studies (*n* = 18) with mercury (Hg) levels (blood and hair, total Hg and/or MeHg) in the general population in different EU countries and the DEMOCOPHES harmonized study in child–mother pairs (hair, total Hg) in 17 EU countries as a reference. Two approaches were included in the RA strategy: the first one was based on estimations of the fraction of children/adolescents and women of childbearing age, respectively, from the EU general population exceeding the HBM-I value established by the German Human Biomonitoring Commission, measured as Hazard Quotients (HQ); and the second approach was based on estimations of the fraction of the two population groups exceeding the Tolerable Weekly Intake (TWI) (or their equivalent to Tolerable Daily Intake (TDI)) defined by EFSA in 2012. The HQ approach showed that for both groups, the risk varies across EU countries and that some EU areas are close to or exceeding the exposure guidance values. This is the case of Spain and Portugal, which showed the highest HQ (GM and/or P95), probably due to their higher fish consumption. Results from the EFSA approach show that hair values of children/adolescents and women of childbearing age (both in selected HBM studies and in DEMOCOPHES study) are below the TDI of 1.9 µg/g; therefore, in general, the European population does not exceed the daily average/intake dose for MeHg and/or Hg. A possible risk underestimation was identified in our assessment since for many studies no data on P95 were available, causing loss of relevant information for risk characterization on the upper bound. In addition, data from other European countries also with high seafood consumption, such as France, Greece or Iceland, were not available. For this reason, further RA refinement is needed with harmonized and more widespread HBM data to account for differences in European exposure and associated risks, so that interventions to protect vulnerable citizens, can be applied.

## 1. Introduction

Mercury (Hg) is a highly toxic heavy metal that poses a significant global threat to human beings and the environment. Together with its various compounds, it can cause severe impacts on human health, including irreversible damage to the central nervous system [1].

Elemental Hg is released into the atmosphere from many natural and anthropogenic sources. Once released, Hg can move around the globe and can remain in circulation for thousands of years. Mercury in water bodies presents the greatest risk to humans because it is converted by microorganisms into methylmercury (MeHg), which is the most toxic form of this metal, easily absorbed by animals and bioaccumulated throughout the food chain [2]. Among the MeHg effects are irreversible damage to the central nervous system, even at very low levels depending on the age exposure window. Fetuses, newborns and children are amongst the most vulnerable and sensitive to the adverse neurodevelopmental effects caused by environmental MeHg exposure. Other relevant population groups are adolescents, as they are completing their developmental period, and pregnant women or women of childbearing age, due to the potential transfer to the fetuses (MeHg can cross the placenta and act on the developing nervous system) [2,3,4].

In Europe, the most significant source of human exposure to MeHg is the diet, especially in populations with high seafood consumption [3], such as in coastal regions—the Mediterranean region of Europe or Arctic region. Exposure levels are also influenced by the type of fish consumed (eating high predatory fish entails a higher risk), as well as their capture areas.

The U.S. Environmental Protection Agency (EPA), the World Health Organization (WHO) and the European Food Safety Authority (EFSA) [4,5,6,7], have compiled the adverse effects of MeHg in humans, including neurocognitive, motoric, auditory and visual electrophysiological responses, as well as neurobehavioral effects. Pivotal studies for dose-response analysis were derived from the cohorts in the Faroe Islands where whale meat is predominant in the diet [8,9] and in Seychelles with a high fish diet [10]. Different guidance values have been established in order to protect human health, having as point of departure (PoD) the findings observed in the aforementioned cohorts. EFSA set a tolerable weekly intake (TWI) for MeHg of 1.3 μg/kg of body weight [4]. The Human Biomonitoring Commission of the German Environment Agency derived Human Biomonitoring (HBM) values for total Hg in blood for children and adults of 5 µg/L(HBM-I) and 15 µg/L (HBM-II), respectively [11].

In the Minamata Convention on Mercury [12], measures were adopted to reduce global Hg emissions. Due to the long-range Hg transport and its bioaccumulation and biomagnification in the environment, these reduction measures will not have an immediate result and, therefore, the evaluation of the current exposure to MeHg in the general population and especially in vulnerable populations continues to be important [13]. This can be performed by estimating the external exposure to Hg, through the determination of the concentration of Hg in specific matrices such as food, water and air [14]; however, this ambient monitoring approach does not provide data about the real uptake by the human body [15].

In this context, human biomonitoring (HBM) is a very useful tool because it provides information on the real internal exposure to Hg. Different biological matrices are used to measure Hg, blood and hair being the most relevant specifically for MeHg, whereas urinary Hg is mainly reflecting exposure to inorganic mercury. Blood Hg levels reflect more recent exposure. The measurement of total Hg in blood reflects exposure to all forms, organic, inorganic and elemental mercury, with MeHg accounting for around 60–91% [16]. Hair is the preferred choice for many studies because it provides a simple, integrative and non-invasive sample for estimating long-term average exposure to MeHg since 89–91% of the total Hg is estimated to be in this organic form [17,18]. Once incorporated in the hair, Hg does not return to the blood; therefore, it provides a good long-term marker of exposure to MeHg [19]. In addition, Hg levels in hair show a direct relationship with blood total Hg levels through the traditional conversion ratio 250:1 established by WHO and recently reviewed and updated to 280:1 by [20].

Although HBM studies are currently recognized as an appropriate tool for exposure and risk assessment [21], their use is still limited in the European risk assessment schemes [22]. In this context, the European Human Biomonitoring Initiative (HBM4EU) has established a European-wide HBM program to generate knowledge on human internal exposure to chemical pollutants and their potential health impacts in Europe, to support policy makers’ efforts to regulate chemical safety and improve public health in Europe (https://www.hbm4eu.eu/ accessed on 17 June 2022) [23].

The aim of this study is to present how European HBM data on Hg can be used in different risk assessment schemes for MeHg in order to estimate the risk for vulnerable European populations and to identify potential challenges.

## 2. Materials and Methods

### 2.1. Study Population and HBM Dataset

Children/adolescents from 3 to 17 years old and women of childbearing age in the range of 18 to 50 years old were selected as relevant population groups for this RA.

A systematic search of available Human Biomonitoring data from EU countries on methylmercury and total mercury was conducted through IPCHEM (The Information Platform for Chemical Monitoring), which is the European Commission’s reference access point for searching, accessing and retrieving chemical occurrence data collected and managed in Europe, and based on the HBM4EU Scoping Document [24], for the two population groups described above. In addition, SCOPUS, PubMed and Web of Science were consulted (accessed on 9 March 2022). The criteria for selecting specific HBM datasets were the following: 1) studies with available data of Hg/MeHg levels in hair or blood expressed as geometric mean (GM) and/or 95th percentile (P95), and 2) with participants from at least one of the two population groups of interest, as defined previously. The bibliographic search strategy is detailed in the Supplementary Materials.

### 2.2. RA Based on Integrating Data from European HBM Surveys

The methodology used in the current MeHg RA is based on the risk-based approach for non-cancer effects published by St-Amand et al. [25] and recently verified by [26]. For non-cancer endpoints, hazard quotients (HQ) are calculated as the ratio of the biomarker concentration to the chemical specific Biomonitoring Equivalents (BE) or Human Biomonitoring Guidance Values (HBM-GVs) (Equation (1)):

(1) $$\mathrm{HQ}=\frac{\left[\mathrm{Biomarker}\right]}{\mathrm{BE}\;\mathrm{or}\;\mathrm{HBM}-\mathrm{GV}}\;$$

where biomarker concentration, [biomarker], is the geometric mean (GM) or upper bound (P95 in this case, reflecting the reasonable worst-case estimate). BEs are based on reference dose (RfD) or tolerable daily intake (TDI) values. As there are no BEs for Hg and, thus, for MeHg, the HBM-GVs derived for Hg by the German HBM Commission [11] were used for HQ calculation. In this case, the HBM-I value for total Hg in blood, 5 µg/L, was selected for evaluation of the risk, since it is the most restrictive HBM-GV and since blood can be used to document recent exposure to MeHg. Those studies in which Hg data (GM and/or P95) were only available in hair, were converted to blood levels using the hair to blood conversion ratio of 280:1 [20].

### 2.3. RA Based on EFSA (2012) Approach

The EFSA Panel on Contaminants in the Food Chain (CONTAM Panel) delivered a scientific opinion on the risks to human health related to the presence of inorganic mercury and MeHg in food in 2012 [4], establishing a tolerable weekly intake (TWI) for MeHg of 1.3 μg/kg body weight (b.w.), expressed as Hg. In order to evaluate the chronic exposure to MeHg, we compared this value to the Hg concentrations found in the selected HBM surveys. For this, a conversion of TWI to hair content according to the WHO [19] was carried out. It is estimated that a daily MeHg average intake of 0.1 µg per kg body weight per day (0.1 μg/kg b.w./d) by an adult woman results in hair Hg concentrations of about 1 μg/g and this relationship is generally directly proportional [19]. Thereby, a TWI of 1.3 µg/kg b.w for MeHg, equivalent to a TDI of 0.19 µg/kg b.w./d, corresponds to hair levels of 1.9 µg/g. This derived value of 1.9 µg/g was compared to exposure levels (total Hg in hair) in the two study population groups, children/adolescents and women of childbearing age from the EU general population.

## 3. Results and Discussion

### 3.1. HBM Dataset

**Figure 1:**
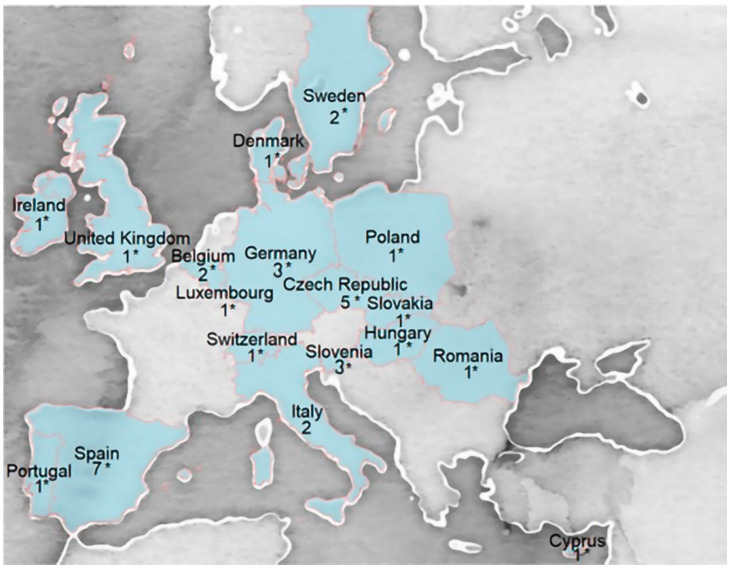
Distribution of European HBM studies included in the MeHg risk assessment. * Indicates countries included in the DEMOCOPHES study. Numbers in the figure indicate the number of studies in each country.

A total of thirty-five European HBM studies were identified fulfilling the above-indicated criteria (available GM and/or P95 hair or blood Hg/MeHg levels in children/adolescents 3–17 years old and/or women of childbearing age 18–50 years old) (Table 1). These included 17 individual DEMOCOPHES studies and 18 additional European HBM studies. Figure 1 shows the geographical distribution of all the studies included in this RA.

The DEMOCOPHES study was used as reference, since it is a well-defined cross-sectional survey of exposure to environmental chemicals, including Hg, performed in 17 European countries using a harmonized European protocol (developed by its twin project, COPHES) [28] and applying strict Quality Control/Quality Assurance (QA/QC) measures in the chemical analysis of the hair samples [46]. In DEMOCOPHES, a total of 1875 child/mother pairs were recruited from urban and rural areas in the participating countries, while excluding exposure hotspots. The exposure biomarkers included the hair Hg concentration (expressed as GM for the individual studies and as GM and P95 for the whole study (DEMOCOPHES-17)) of samples collected from September 2011 to February 2012 from children aged 6–11 years old and their mothers (<45 years old).

Among the international studies retrieved, only two reported levels of MeHg in children and/or women, one in hair [27], and the other one in blood and hair [35], while the remaining studies reported only total Hg levels.

To assess the relevant results obtained across Europe for children/adolescents and women of childbearing age from the 18 European HBM studies recorded in Table 1 (excluding DEMOCOPHES data) and to compare them to the DEMOCOPHES data, a range of GM and a range of P95 of MeHg and/or Hg blood concentrations was compiled (Table 2). As mentioned earlier, for those studies with data for only MeHg and/or Hg concentrations in hair, the hair-to-blood conversion ratio of 280:1 recently proposed by Esteban et al. [20] was used. Previous studies [20,47,48] have shown that there is a high interindividual variation in the calculated hair-to-blood ratios in different populations and even at different ages. In the study by Esteban et al. [20], focused on European populations, the geometric mean hair to blood ratio of 280 predicted blood values equally well to the regression equation of hair Hg vs. blood Hg, while when the ratio of 250 recommended by WHO was used, blood Hg estimations were significantly less accurate from those predicted by the regression equation or by using the 280 ratio.

When conducting the HBM data search, we observed a high degree of heterogeneity in the data available for the different European studies not only related to the biomarkers/matrices measured, but also to the descriptive statistics reported. This limited the analysis, which was mostly performed using GM values, losing risk characterization on the upper bound.

Data from 18 studies, in addition to the 17 included in DEMOCOPHES, were retrieved; this made information available for 18 European countries, although the degree of representativeness of the data collected is limited. In addition, we observed that data from some other European countries with high seafood consumption, such as France, Greece or Iceland, and which could be relevant for the MeHg risk assessment performed here, were not available.

Most of the studies did not perform Hg speciation analysis (Table 1); therefore, we used total Hg concentrations as surrogate because MeHg is the main contributor to the levels found both in blood and hair as reported in the literature [16,17,18]. This was also observed from the studies which had total Hg and MeHg measured [27,35]. However, in the case of the Belgian FLESH study [27], 63% and 74% of the total Hg in hair was in the form of MeHg in adolescents and women, respectively. This could be associated with different factors relative to the region or lifestyle, such as seafood consumption patterns (lower consumption) in these groups. In the study carried out by Valent et al. [35], the percentages seem to be higher although they cannot be calculated as the number of samples in which speciation was done differs from the number in which total Hg was measured.

### 3.2. RA Based on Integrating Data from European HBM Surveys

The HQ were calculated as the ratio between the GM or the P95 of the MeHg and/or Hg blood concentrations retrieved in the selected HBM datasets (Table 2) and the HBM-I value for Hg in blood of 5 µg/L (Equations (2) and (3)), following a conservative approach as mentioned earlier:

(2) $$\mathrm{HQ}\;(\mathrm{GM})=\frac{\mathrm{GM}\;\mathrm{of}\;\mathrm{HBM}\;\mathrm{results}\;\mathrm{distribution}}{\mathrm{Selected}\;\mathrm{internal}\;\mathrm{threshold}\;\left(\mathrm{HBM}-\mathrm{GV}\;\mathrm{of}\;5\;µg/L\right)}$$

(3) $$\mathrm{HQ}\;(P95)=\frac{P95\;\mathrm{of}\;\mathrm{HBM}\;\mathrm{results}\;\mathrm{distribution}}{\mathrm{Selected}\;\mathrm{internal}\;\mathrm{threshold}\;\left(\mathrm{HBM}-\mathrm{GV}\;\mathrm{of}\;5\;\text{µ}g/L\right)}$$

HQs near or exceeding 1 indicate that exposure levels are near or exceeding selected exposure guidance value.

Table 3 shows the HQ calculations for the GM and/or P95 of Hg levels in blood for the range of GM and P95 of all the studies together for the selected populations. Supplementary Table S1 shows the HQs calculated for each study included in this RA.

**Figure 2:**
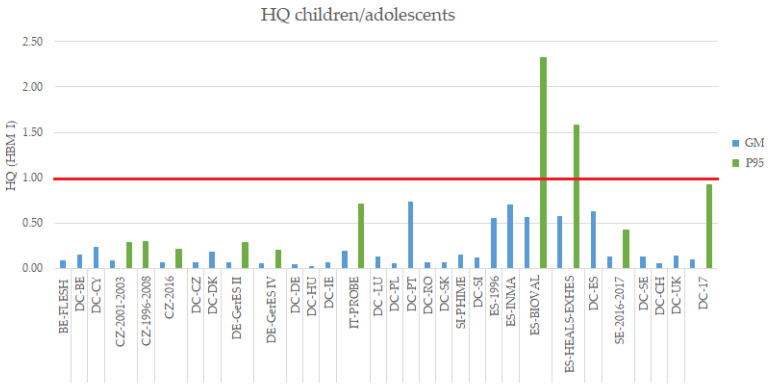
HQ for the GM and P95 of total Hg blood levels of the HBM European studies in children/adolescents (3–17 years old). DEMOCOPHES Study (DC). Note: MeHg levels in the case of FLESH study.

In the case of children/adolescents, the HQs calculated for the range of GMs for the HBM studies included in this work (excluding DEMOCOPHES) do not exceed the value of 1 (Figure 2). However, the P95 for some Spanish studies are over 1 (HQ_P95 HEALS-EXHES_ = 1.58 and HQ_P95 BIOVAL_ = 2.32, Supplementary Table S1).

The HQs for DEMOCOPHES study, both for the range of GMs of the 17 individual studies, as well as for the GM of the study as a whole, do not exceed the value of 1(Figure 2). However, the P95 of DEMOCOPHES-17 is close to the value of 1 (HQ_P95 DEMOCOPHES-17_ = 0.92). The GM of other individual countries, for example Portugal, are close (HQ_GM DEMOCOPHES-PT_ = 0.74).

**Figure 3:**
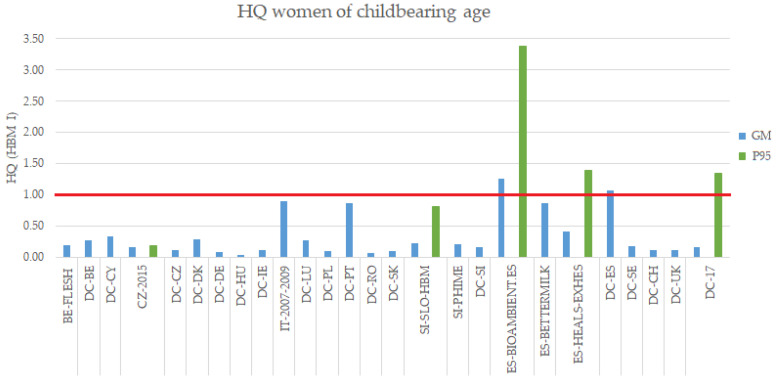
HQ for the GM and P95 of Hg blood levels of the HBM European studies in women of childbearing age (18–50 years old). DEMOCOPHES Study (DC). Note: MeHg levels in the case of FLESH and Valent et al., 2013 [35] studies.

For women of childbearing age, the HQ calculated for the range of GMs and P95 in the selected European HBM studies (excluding DEMOCOPHES) exceed the value of 1 only in two studies from Spain (HQ_GM BIOAMBIENT.ES-SP_ = 1.25 and HQ_P95 BIOAMBIENT.ES-SP_ = 3.38; HQ_P95 HEALS_ = 1.40) and one study also from Spain, was close to 1 (HQ_GM BETTERMILK_ = 0.87) (Figure 3).

The HQ for DEMOCOPHES showed that, in contrast to the results observed for the children/adolescents, both the upper part of the range of GMs of the 17 individual studies in DEMOCOPHES, as well as the P95 of the whole study are close or exceeding the value of 1 (HQ_P95 DEMOCOPHES_ = 1.35 and HQ_range GM DEMOCOPHES_ = 0.03–1.06). Spain and Portugal are among the countries with GM exceeding or close to the guidance values (HQ_GM DEMOCOPHES-ES_ = 1.06; HQ_GM DEMOCOPHES-PT_ = 0.86) (Figure 3), the latter being consistent with the findings in the case of children/adolescents.

Overall, risk assessment based on HQ using the HBM-I guidance value showed that the risk varies in the different EU countries and some EU areas are close to or exceeding this exposure guidance value, both for children/adolescents and women of childbearing age. This is the case of Spain and Portugal, which were the countries with highest HQ, probably due to their higher seafood consumption frequency or species consumed, as was already described in DEMOCOPHES [28]. The guidance value selected here was the HBM-I, being a most conservative HBM-GV for evaluating the risk associated to MeHg and/or Hg exposure. At exposure levels exceeding the HBM-I value, but falling below the HBM-II value, adverse health effects cannot be excluded with sufficient certainty and a follow-up examination should be performed to determine whether there is a continued elevated exposure [19]. Since the main MeHg exposure source is seafood consumption, the recommendation to minimize risk would be to develop harmonized dietary advice. This would allow benefitting from the seafood consumption while protecting the health of vulnerable populations, such as pregnant women and children. Recommendations on how to reduce the uptake of MeHg were published by EFSA in 2015 [5]. However, due to the variety of fish species consumed across Europe, EFSA’s Scientific Committee suggested that it is not possible to make general recommendations and that the situation should be evaluated in each country. Most European countries, however, do not have official recommendations on the fish consumption. The effectiveness of the proper implementation of dietary advice to pregnant women to lower mercury intake has been demonstrated in a recent study in Denmark [49]. More recently, it has also been addressed in the HBM4EU-MOM study, where a harmonized intervention focusing on dietary advice to reduce prenatal exposure to mercury in 5 European coastal countries has been carried out [50]. Proper and harmonized communication of these findings to the European population, and particularly to vulnerable groups, is paramount.

In the DEMOCOPHES study, we have observed that the ranges of GMs and P95 were smaller in comparison to those obtained in the rest of European datasets, with a similar geographical distribution. This could be due to the fact that DEMOCOPHES used a harmonized protocol for all participating countries and the rest of European studies have followed different protocols. However, it is necessary to point out that for the individual DEMOCOPHES studies and some other HBM studies in this RA, no P95 values were available, and the overall risk characterization had to be performed mostly using GM.

Furthermore, the publicly available HBM data do not include information from several European countries with high seafood consumption, such as other Mediterranean countries, which could probably affect significantly the results obtained.

### 3.3. RA Based on EFSA (2012) Approach

Exposure levels (Hg in hair, GM and P95) in children/adolescents and women of childbearing age from the EU general population were compared with the available exposure guideline value for Hg in hair of 1.9 µg/g derived here, obtained after transformation of the TWI for MeHg of 1.3 µg/kg b.w established by EFSA (detailed in Section 2.3).

**Figure 4:**
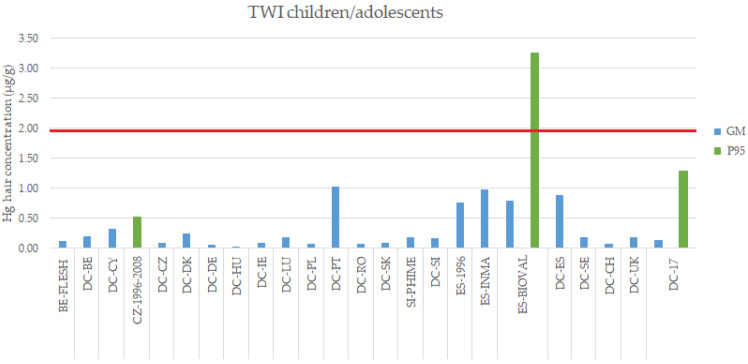
Exposure levels (Hg in hair, GM and P95) in children/adolescents (3–17 years old) from the EU general population compared to the internal exposure guideline value for Hg in hair derived from EFSA (1.9 µg/g, in red). DEMOCOPHES Study (DC). Note: MeHg levels in the case of FLESH study.

In the case of the children/adolescents, only one study exceeded the 1.9 µg/g of Hg in hair, namely the BIOVAL program, conducted in Spain in 2016, with a P95 value of 3.25 µg/g (Figure 4). However, when evaluating GM values, no study exceeded this value; therefore, in terms of daily average or intake dose, the recommended values by EFSA are not being exceeded.

**Figure 5:**
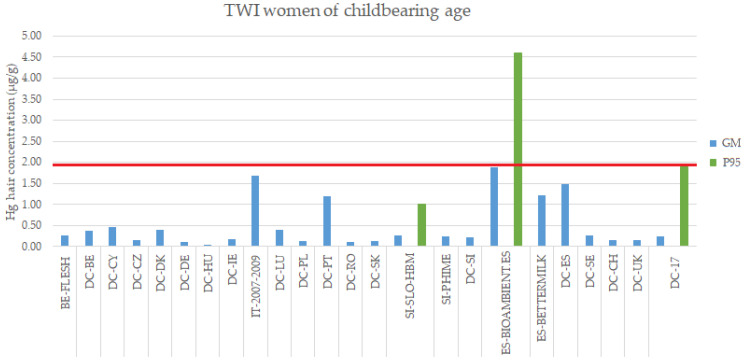
Exposure levels (Hg in hair, GM and P95) in women of childbearing age (18–50 years old) from the EU population compared to the internal exposure guideline value for Hg in hair derived from EFSA (1.9 µg/g in red). DEMOCOPHES Study (DC). Note: MeHg levels in the case of FLESH and Valent et al., 2013 [35] studies.

In the case of women (Figure 5), only one study from Spain, BIOAMBIENT.ES, had Hg hair levels close to or over the 1.9 µg/g reference value with a GM of 1.87 µg/g and a P95 value of 4.6 µg/g, respectively. Amongst the other studies, those with GM closer to the reference value included a study carried out in pregnant women in Italy with a hair MeHg GM value of 1.67 µg/g [35], further two studies in Spain, DEMOCOPHES-ES (GM of 1.49 µg/g) and BETTERMILK project (GM of 1.22 µg/g), and DEMOCOPHES-PT in Portugal (GM of 1.20 µg/g). As indicated earlier, there were no data on P95 values for any of these studies, which hampers the risk assessment as these are all countries with high seafood consumption, and with higher GM values, and could potentially exceed the HQ of 1 if the risk assessment is based on P95 levels representing reasonable worst-case scenario. This research confirms previous findings for five European countries (Belgium, Ireland, Italy, Portugal and Spain), that showed that larger subgroups of the population in the southern European countries (up to 11% in Portugal) were potentially at risk for a MeHg exposure above the TWI value, while this risk was much lower in Ireland and Belgium [51]. Nevertheless, in general terms, the European population, as represented by the studies included in this RA, does not exceed the daily average/intake dose for MeHg and/or Hg.

Most European HBM studies included in this RA measured total Hg in blood and/or in hair, and only two studies analyzing the different Hg species, were identified. Speciation analysis is necessary to differentiate between inorganic/elemental Hg and MeHg exposure. However, speciation of Hg requires complex and lengthy analytical procedures and expensive reagents and equipment, which are not routinely available in analytical laboratories. Furthermore, in the case of hair, it is accepted that total Hg analysis can be used as surrogate of MeHg analysis, due to the high percentage (89–91%) of this form in the total Hg concentration found in hair; therefore, it is common that HBM studies measure total Hg in hair [20]. In addition, as mentioned earlier, it is also recognized that MeHg accounts for a high proportion (60–91%) of the total Hg in blood in general population without, for example, occupational exposure to inorganic Hg [16]. The development and implementation of harmonized methodologies would allow an adequate characterization of the exposure to each type of Hg in vulnerable populations in Europe. Therefore, the appropriate selection of the matrices and biomarkers of exposure is crucial to reduce the potential uncertainties associated to the conversion rates in the risk assessment [20].

## 4. Conclusions

The followed HQ approach showed that for both children/adolescents and women of childbearing age, there is a high variability in the risk across EU countries. EU areas with high seafood consumption (Spain and Portugal) showed the highest HQ, close to or exceeding the exposure guidance value. Using the approach based on the TDI derived here in both studied populations, hair values were below TDI of 1.9 µg/g, suggesting that the European population does not generally exceed the daily average/intake dose for MeHg and/or Hg. However, since some countries may be at higher risk due to their diet and that climate change can increase the MeHg levels in fish [52], efforts to develop dietary recommendations focusing on the vulnerable populations should be planned and implemented. This would be especially important in Mediterranean countries with fish consumption patterns that, as seen here, can lead to HQ values above 1.

The HBM approach provides a direct way to assess the factors contributing to the risk and to take actions for risk communication and management in a more appropriate and personalized way, as needed.

The work reported here highlights the need for further harmonization in HBM studies. Although a significant effort has been made to study the real exposure to MeHg of vulnerable populations throughout Europe, the lack of harmonization in the HBM studies, both in terms of the biomarkers used and the descriptive statistics reported, has limited the risk assessment in this case. The availability of data on relevant determinants, e.g., seafood consumption, and, ideally, on health outcomes would be of importance for a more comprehensive analysis, in order to assess health risk resulting from Hg and/or MeHg exposure

In addition, a possible data underestimation was identified as a limitation, since for many studies the P95 data were not available and, thereby, losing an important information of the upper bound for risk characterization. In addition, HBM data from other European countries with high fish consumption, such as France, Greece or Iceland, were not available. For this reason, as a recommendation, further RA refinement is needed with more widespread harmonized HBM data to account for differences in European exposure.

## Figures and Tables

**Table 1:** Human biomonitoring (HBM) studies with Hg and MeHg data in blood and hair in the selected populations.

Country	Study	Year	Population Age (*N*)	Hg				MeHg				Refs.
				Blood (µg/L)		Hair (μg/g)		Blood (µg/L)		Hair (μg/g)		
				GM	P95	GM	P95	GM	P95	GM	P95	
Belgium	FLESH	2007–2011	Adolescents 14–15 y (206)	−	−	0.19	−	−	−	0.12	−	[27]
			Mothers 20–40 y (242)	−	−	0.35	−	−	−	0.26	−	
	DEMOCOPHES-BE	2010–2012	Children 6–11 y (127)	−	−	0.20	−	−	−	−	−	[28]
			Mothers <45 y (127)	−	−	0.37	−	−	−	−	−	
Cyprus	DEMOCOPHES-CY	2010–2012	Children 6–11 y (60)	−	−	0.33	−	−	−	−	−	[28]
			Mothers <45 y (60)	−	−	0.46	−	−	−	−	−	
Czech Republic	CZ-HBM	2001–2003	Children 8–10 y (333)	0.43	1.44	−	−	−	−	−	−	[29]
	CZ-HBM	1996–2008	Children 8–10 y (344 ^a^/347) *		1.47		0.52	−	−	−	−	[30]
	DEMOCOPHES-CZ	2010–2012	Children 6–11 y (120)	−	−	0.10	−	−	−	−	−	[28]
			Mothers <45 y (120)	−	−	0.16	−	−	−	−	−	
	CZ-HBM	2015	Women 18–50 y (n.a.)	0.80	0.90	−	−	−	−	−	−	[31]
	CZ-HBM	2016	Children 5 & 9 y (419)	0.32	1.03	−	−	−	−	−	−	[32]
Denmark	DEMOCOPHES-DK	2010–2012	Children 6–11 y (144)	−	−	0.25	−	−	−	−	−	[28]
			Mothers <45 y (144)	−	−	0.39	−	−	−	−	−	
Germany	GerES II	1990–1992	Children 6–17 y (712)	0.33	1.40	−	−	−	−	−	−	[33]
	GerES IV	2003–2006	Children 3–14 y (1240)	0.23	1.00	−	−	−	−	−	−	[33]
	DEMOCOPHES-DE	2010–2012	Children 6–11 y (120)	−	−	0.06	−	−	−	−	−	[28]
			Mothers <45 y (120)	−	−	0.11	−	−	−	−	−	
Hungary	DEMOCOPHES-HU	2010–2012	Children 6–11 y (119)	−	−	0.03	−	−	−	−	−	[28]
			Mothers <45 y (119)	−	−	0.04	−	−	−	−	−	
Ireland	DEMOCOPHES-IE	2010–2012	Children 6–11 y (120)	−	−	0.10	−	−	−	−	−	[28]
			Mothers <45 y (120)	−	−	0.16	−	−	−	−	−	
Italy	PROBE	2008–2010	Adolescents 13–15 y (252)	0.94	3.55	−	−	−	−	−	−	[34]
	−	2007–2009	Pregnant women n.a. (606 ^a^/604 ^b^/236 ^c^/220 ^d^)	3.14	−	1.06	−	4.46	−	1.67	−	[35]
Luxembourg	DEMOCOPHES -LU	2010–2012	Children 6–11 y (56)	−	−	0.18	−	−	−	−	−	[28]
			Mothers <45 y (56)	−	−	0.39	−	−	−	−	−	
Poland	DEMOCOPHES-PL	2010–2012	Children 6–11 y (120)	−	−	0.07	−	−	−	−	−	[28]
			Mothers <45 y (120)	−	−	0.14	−	−	−	−	−	
Portugal	DEMOCOPHES-PT	2010–2012	Children 6–11 y (120)	−	−	1.03	−	−	−	−	−	[28]
			Mothers <45 y (120)	−	−	1.20	−	−	−	−	−	
Romania	DEMOCOPHES-RO	2010–2012	Children 6–11 y (120)	−	−	0.09	−	−	−	−	−	[28]
			Mothers <45 y (120)	−	−	0.10	−	−	−	−	−	
Slovakia	DEMOCOPHES-SK	2010–2012	Children 6–11 y (129)	−	−	0.09	−	−	−	−	−	[28]
			Mothers <45 y (129)	−	−	0.13	−	−	−	−	−	
Slovenia	SLO-HBM	2008–2009, 2011–2014	Women 19–39 y (535 ^a^/503 ^b^)	1.1	4.06	0.27	0.99	−	−	−	−	[36]
	PHIME project	2011–2014	Children 6–11 y (174)	0.77	−	0.18	−	−	−	−	−	[37]
			Women 20–35 y (127)	1.04	−	0.24	−	−	−	−	−	
	DEMOCOPHES-SI	2010–2012	Children 6–11 y (120)	−	−	0.17	−	−	−	−	−	[28]
			Mothers <45 y (120)	−	−	0.23	−	−	−	−	−	
Spain	−	1996	Children 6–16 y (233)	−	−	0.77	−	−	−	−	−	[38]
	BIOAMBIENT.ES	2009–2010	Women >18 y (918 ^a^/327 ^b^)	6.27	16.90	1.87	4.6	−	−	−	−	[39]
	INMA Project	2008–2012	Children 4–5 y (1252)	−	−	0.98	−	−	−	−	−	[40]
	DEMOCOPHES-ES	2010–2012	Children 6–11 y (120)	−	−	0.88	−	−	−	−	−	[28]
			Mothers <45 y (120)	−	−	1.49	−	−	−	−	−	
	BIOVAL programme	2016	Children 6–11 y (611)	−	−	0.79	3.25	−	−	−	−	[41]
	BETTERMILK Project	2017	Breastfeeding mothers 20–45 y (120)	−	−	1.22	−	−	−	−	−	[42]
	HEALS-EXHES	2016–2017	Children cord blood (53)	2.87	7.91	−	−	−	−	−	−	[43,44]
			Mothers GM 34 y (53)	2.05	6.98	−	−	−	−	−	−	
Sweden	DEMOCOPHES-SE	2010–2012	Children 6–11 y (100)	−	−	0.18	−	−	−	−	−	[28]
			Mothers <45 y (100)	−	−	0.25	−	−	−	−	−	
	−	2016–2017	Children/adolescents 12, 15 & 18 y (1099)	0.66	2.10	−	−	−	−	−	−	[45]
Switzerland	DEMOCOPHES-CH	2010–2012	Children 6–11 y (120)	−	−	0.08	−	−	−	−	−	[28]
			Mothers <45 y (120)	−	−	0.15	−	−	−	−	−	
United Kingdom	DEMOCOPHES-UK	2010–2012	Children 6–11 y (21)	−	−	0.19	−	−	−	−	−	[28]
			Mothers <45 y (21)	−	−	0.15	−	−	−	−	−	
17 EU countries	DEMOCOPHES-17	2010–2012	Children 6–11 y (1836)	−	−	0.14	1.29	−	−	−	−	[28]
			Mothers <45 y (1839)	−	−	0.23	1.89	−	−	−	−	

^a^*N* for total Hg in blood. ^b^ *N* for total Hg in hair. ^c^ *N* for MeHg in blood. ^d^ *N* for MeHg in hair. * Missing years: Hg in blood 1997, 2000, 2002, 2003, 2004, 2005, 2007; Hg in hair 2004, 2005, 2007. n.a. not available.

**Table 2:** Range of geometric means (GM) and/or 95th percentiles (P95) of MeHg and/or Hg concentrations in blood (µg/L) (hair values are expressed as blood concentration using hair to blood ratio of 280:1) from European HBM studies and GM and P95 values for the whole DEMOCOPHES-17 reference study.

	DEMOCOPHES		Other HBM Studies	
	Children	Mothers	Children/Adolescents	Women
	(17 Studies)	(17 Studies)	(13 Studies)	(9 Studies)
**Range of GM**	0.09–3.69	0.14–5.31	0.23–3.50	0.80–6.27
**Range of P95**	−	−	1.03–11.6	0.90–16.9
**GM_DEMOCOPHES-17_**	0.51	0.82	−	−
**P95_DEMOCOPHES-17_**	4.60	6.75	−	−

**Table 3:** HQ calculations for the GM and/or P95 of Hg levels in blood for all HBM studies considered for the selected populations.

	Children/Adolescents		Women of Childbearing Age	
	European HBM Studies	DEMOCOPHES	European HBM Studies	DEMOCOPHES
**HQ_range GM_**	0.05 to 0.70	0.02 to 0.74	0.16 to 1.25	0.03 to 1.06
**HQ_range P95_**	0.21 to 2.32	−	0.18 to 3.38	−

## Data Availability

Aggregate data included in this study were publicly available from IPCHEM (https://ipchem.jrc.ec.europa.eu/, accessed on 9 March 2022), SCOPUS (https://www.scopus.com, accessed on 9 March 2022), PubMed (https://pubmed.ncbi.nlm.nih.gov, accessed on 9 March 2022) and Web of Science (institutional access through the Fundación Española para la Ciencia y Tecnología, Spanish Ministry of Science and Innovation). These sites were last accessed on 9 March 2022.

## Supplementary Materials

The following supporting information can be downloaded at: https://www.mdpi.com/article/10.3390/toxics10080427/s1, Search strategy on the bibliographic databases and Table S1: Hazard Quotients calculated for each study included in this risk assessment.
