# Supplementary material for: Methylmercury Risk Assessment Based on European Human Biomonitoring Data
Source: Toxics. 2022 Jul 28;10(8):427. doi: 10.3390/toxics10080427 (PMC9416112; doi:10.3390/toxics10080427)
Supplement: Supplementary file 1 [file toxics-10-00427-s001.zip › toxics-1799145-supplementary.pdf]

toxics

MDPI

SUPPLEMENTARY MATERIAL

Methylmercury Risk Assessment based on European Human Biomonitoring Data

Noelia Domínguez-Morueco [1], Susana Pedraza-Díaz [1*], M. Carmen González-Caballero[1], Marta Esteban-López[1], Mercedes de Alba-Gonzalez [1], Andromachi Katsonouri [2], Tiina Santonen [3] Ana Isabel Cañas-Portilla [1*]and Argelia Castaño [1]

[1] National Centre for Environmental Health, Instituto de Salud Carlos III, 28220 Madrid, Spain; ndominguez@isciii.es; spedraza@isciii.es; mcgonzalez@isciii.es; m.esteban@isciii.es; malba@isciii.es; acanas@isciii.es; castano@isciii.es

[2] Cyprus State General Laboratory, Ministry of Health, P.O. Box 28648, 2081 Nicosia, Cyprus; akatsonouri@sgl.moh.gov.cy

[3] Finnish Institute of Occupational Health, P.O. Box 40, FI-00032, Työterveyslaitos, Finland, tiina.santonen@ttl.fi

* Correspondence: spedraza@isciii.es; acanas@isciii.es

SUPPLEMENTARY MATERIAL

Bibliographic search strategy

A systematic search of available Human Biomonitoring data from EU countries on methylmercury and total mercury was conducted through the IPCHEM (The Information Platform for Chemical Monitoring), which is the European Commission's reference access point for searching, accessing and retrieving chemical oc-currence data collected and managed in Europe and in the HBM4EU Scoping Document [24]. The search keywords used here were: "Mercury" AND "Human Biomonitoring Data" and "Methylmercury" AND "Human Biomonitoring Data".

In addition, SCOPUS, PubMed and Web of Science were consulted (accessed on 9 March 2022). The search keywords used were: "Methylmercury" OR "Mercury" AND "Human Biomonitoring"; "Methylmercury" OR "Mercury" AND "Blood Samples"; "Methylmercury" OR "Mercury" AND "Hair Samples"; "Methylmercury" OR "Mercury" AND "Blood"; "Methylmercury" OR "Mercury" AND "Hair" "Methylmercury" OR "Mercury" AND "Children"; "Methylmercury" OR "Mercury" AND "Adolescents"; "Methylmercury" OR "Mercury" AND "Woman of childbearing age"; " Mercury Human Biomonitoring" AND "European population". After primary screening to remove the articles with irrelevant topics, a secondary screening was done to select the articles with the following specific HBM datasets 1) Studies with available data of Hg and/or MeHg levels in hair or blood expressed as geometric mean (GM) and/or 95th percentile (P95) and 2) with participants from at least one of the two population groups of interest, as defined previously.

Table S1. HQ calculated for each study included in this RA.

Country	Study	Population (N)	Total Mercury Blood (μg/L) GM	Total Mercury Blood (μg/L) P95	Methylmercury Blood (μg/L) GM	Methylmercury Blood (μg/L) P95	Total Mercury HQ (HBM I) GM	Total Mercury HQ (HBM I) P95	Methylmercury HQ (HBM I) GM	Methylmercury HQ (HBM I) P95	Ref.
Belgium	FLESH	Adolescents 14-15y (206)	0.68	–	0.43	–	0.14	–	0.09	–	[27]
		Mothers 20-40y (242)	1.25	–	0.93	–	0.25	–	0.19	–	
	DEMOCOPHES-BE	Children 6-11y (127)	0.73	–	–	–	0.15	–	–	–	[28]
		Mothers <45y (127)	1.31	–	–	–	0.26	–	–	–	
Cyprus	DEMOCOPHES-CY	Children 6-11y (60)	1.16	–	–	–	0.23	–	–	–	[28]
		Mothers Age <45y (60)	1.65	–	–	–	0.33	–	–	–	
Czech Republic	CZ-HBM-2001-2003	Children Age: 8-10y (333)	0.43	1.44	–	–	0.09	0.29	–	–	[29]
	CZ-HBM-2008	Children 8-10y (344)	–	1.47	–	–	–	0.29	–	–	[30]
	DEMOCOPHES-CZ	Children 6-11y (120)	0.35	–	–	–	0.07	–	–	–	[28]
		Mothers <45y (120)	0.56	–	–	–	0.11	–	–	–	
	CZ-HBM-2015	Women 18-50 y (n.a.)	0.80	0.90	–	–	0.16	0.18	–	–	[31]
	CZ-HBM-2016	Children 5 & 9y (419)	0.32	1.03	–	–	0.06	0.21	–	–	[32]

Country	Study	Population (N)	Total Mercury Blood (µg/L)		Methylmercury Blood (µg/L)		Total Mercury HQ (HBM I)		Methylmercury HQ (HBM I)		Ref.
			GM	P95	GM	P95	GM	P95	GM	P95	
Denmark	DEMOCOPHES-DK	Children 6-11y (144)	0.89	–	–	–	0.18	–	–	–	[28]
		Mothers <45y (144)	1.40	–	–	–	0.28	–	–	–	
Germany	GerES II	Children 6-17 y (712)	0.33	1.40	–	–	0.07	0.28	–	–	[33]
	GerES IV	Children 3-14 y (1240)	0.23	1.00	–	–	0.05	0.20	–	–	
	DEMOCOPHES-DE	Children 6-11y (120)	0.20	–	–	–	0.04	–	–	–	[28]
		Mothers <45y (120)	0.38	–	–	–	0.08	–	–	–	
Hungary	DEMOCOPHES-HU	Children 6-11y (119)	0.09	–	–	–	0.02	–	–	–	[28]
		Mothers <45y (119)	0.14	–	–	–	0.03	–	–	–	
Ireland	DEMOCOPHES-IE	Children 6-11y (120)	0.35	–	–	–	0.07	–	–	–	[28]
		Mothers <45y (120)	0.58	–	–	–	0.12	–	–	–	
Italy	PROBE	Adolescents 13–15y (252)	0.94	3.55	–	–	0.19	0.71	–	–	[34]
Italy	-	Pregnant women ($606^{Hg}/236^{MeHg}$)	3.14	–	4.46	–	0.63	–	0.89	–	[35]
Luxembourg	DEMOCOPHES -LU	Children 6-11y (56)	0.65	–	–	–	0.13	–	–	–	[28]

Country	Study	Population (N)	Total Mercury Blood (μg/L)		Methylmercury Blood (μg/L)		Total Mercury HQ (HBM I)		Methylmercury HQ (HBM I)		Ref.
			GM	P95	GM	P95	GM	P95	GM	P95	
		Mothers <45y (56)	1.38	–	–	–	0.28	–	–	–	
Poland	DEMOCOPHES-PL	Children 6-11y (120)	0.25	–	–	–	0.05	–	–	–	[28]
		Mothers <45y (120)	0.48	–	–	–	0.10	–	–	–	
Portugal	DEMOCOPHES-PT	Children 6-11y (120)	3.69	–	–	–	0.74	–	–	–	[28]
		Mothers <45y (120)	4.29	–	–	–	0.86	–	–	–	
Romania	DEMOCOPHES-RO	Children 6-11y (120)	0.30	–	–	–	0.06	–	–	–	[28]
		Mothers <45y (120)	0.36	–	–	–	0.07	–	–	–	
Slovakia	DEMOCOPHES-SK	Children 6-11y (129)	0.33	–	–	–	0.07	–	–	–	[28]
		Mothers <45y (129)	0.47	–	–	–	0.09	–	–	–	
Slovenia	SLO-HBM	Women 19-39y (535)	1.10	4.06	–	–	0.22	0.81	–	–	[36]
	PHIME project	Children 6-11y (174)	0.77	–	–	–	0.15	–	–	–	[37]
		Women 20-35y (127)	1.04	–	–	–	0.21	–	–	–	
	DEMOCOPHES-SI	Children 6-11y (120)	0.60	–	–	–	0.12	–	–	–	[28]
	DEMOCOPHES-SI	Mothers <45y (120)	0.80	–	–	–	0.16	–	–	–	

Country	Study	Population (N)	Total Mercury Blood (µg/L)		Methylmercury Blood (µg/L)		Total Mercury HQ (HBM I)		Methylmercury HQ (HBM I)		Ref.
			GM	P95	GM	P95	GM	P95	GM	P95	
**Spain**	-	Children 6-16y (233)	2.75	–	–	–	0.55	–	–	–	[38]
	BIOAMBIENT.ES	Women >18 y (918)	6.27	16.90	–	–	1.25	3.38	–	–	[39]
	INMA Project	Children 4-5y (1252)	3.50	–	–	–	0.70	–	–	–	[40]
	DEMOCOPHES-ES	Children 6-11y (120)	3.16	–	–	–	0.63	–	–	–	[28]
		Mothers <45y (120)	5.31	–	–	–	1.06	–	–	–	
	BIOVAL programme	Children 6-11y (611) Valencia Region	2.82	11.61	–	–	0.56	2.32	–	–	[41]
	BETTERMILK Project	Breastfeeding mothers 20-45y (120)	4.36	–	–	–	0.87	–	–	–	[42]
	HEALS-EXHES	Children cord blood (53)	2.87	7.91	–	–	0.57	1.58	–	–	[43, 44]
		Mothers = 35 GM y (53)	2.05	6.98	–	–	0.41	1.40	–	–	
Sweden	DEMOCOPHES-SE	Children 6-11y (100)	0.65	–	–	–	0.13	–	–	–	[28]
		Mothers <45y (100)	0.90	–	–	–	0.18	–	–	–	
	-	Children/adolescent 12, 15 and 18y (1099)	0.66	2.10	–	–	0.13	0.42	–	–	[45]
Switzerland	DEMOCOPHES-CH	Children 6-11y (120)	0.27	–	–	–	0.05	–	–	–	[28]

Country	Study	Population (N)	Total Mercury Blood (μg/L)		Methylmercury Blood (μg/L)		Total Mercury HQ (HBM I)		Methylmercury HQ (HBM I)		Ref.
			GM	P95	GM	P95	GM	P95	GM	P95	
		Mothers <45y (120)	0.55	–	–	–	0.11	–	–	–	
United Kingdom	DEMOCOPHES-UK	Children 6-11y (21)	0.69	–	–	–	0.14	–	–	–	[28]
		Mothers <45y (21)	0.55	–	–	–	0.11	–	–	–	
17 EU countries	DEMOCOPHES	Children 6-11y (1836)	0.51	4.60	–	–	0.10	0.92	–	–	[28]
		Mothers <45y (1839)	0.82	6.75	–	–	0.16	1.35	–	–	

References

1. Schoeters G., Colles A., Hond E. D., Croes K., Vrijens J., Baeyens W., Nelen V., Mieroop E. V.D., Covaci A., Bruckers L.,Larebeke N.V., Sioen I., Morrens B., Loots I. 2012. Chapter 2F The Flemish Environment and Health Study (FLEHS) - Second Survey (2007-2011): Establishing Reference Values for Biomarkers of Exposure in the Flemish Population. In Biomarkers and Human Biomonitoring: Volume 1; The Royal Society of Chemistry, Vol. 1, pp. 135–165.

2. Den Hond E, Govarts E, Willems H, et al. First steps toward harmonized human biomonitoring in Europe: demonstration project to perform human biomonitoring on a European scale. *Environ Health Perspect.* **2015**, 123(3):255-263. https://doi:10.1289/ehp.1408616

3. Batáriová A, Spevácková V, Benes B, Cejchanová M, Smíd J, Cerná M. Blood and urine levels of Pb, Cd and Hg in the general population of the Czech Republic and proposed reference values. *Int J Hyg Environ Health.* **2006**, 209(4):359-366. https://doi:10.1016/j.ijheh.2006.02.005

4. Puklová V, Krsková A, Cerná M, et al. The mercury burden of the Czech population: An integrated approach. *Int J Hyg Environ Health*. **2010,** 213(4):243-251. https://doi.org/10.1016/j.ijheh.2010.02.002

5. Environmental Health Monitoring System in the Czech Republic. Summary Report, 2015. TIGIS. 1st edition, 96 pages. Prague, September 2016, ISBN 978-80-7071-352-5.

6. Environmental Health Monitoring System in the Czech Republic. Summary Report, 2016. TIGIS. 1st edition, 97 pages. Prague, September 2017, ISBN 978-80-7071-365-5.

7. Schulz C, Conrad A, Becker K, Kolossa-Gehring M, Seiwert M, Seifert B. Twenty years of the German Environmental Survey (GerES): human biomonitoring--temporal and spatial (West Germany/East Germany) differences in population exposure. *Int J Hyg Environ Health*. **2007**, 210(3-4):271-297. https://doi:10.1016/j.ijheh.2007.01.034.

8. Pino A, Amato A, Alimonti A, Mattei D, Bocca B. Human biomonitoring for metals in Italian urban adolescents: data from Latium Region. *Int J Hyg Environ Health*. **2012**, 215(2):185-190. https://doi:10.1016/j.ijheh.2011.07.015

9. Valent F, Mariuz M, Bin M, et al. Associations of prenatal mercury exposure from maternal fish consumption and polyunsaturated fatty acids with child neurodevelopment: a prospective cohort study in Italy. *J Epidemiol.* **2013**, 23(5):360-370. https://doi:10.2188/jea.je20120168

10. Snoj Tratnik J, Falnoga I, Mazej D, et al. Results of the first national human biomonitoring in Slovenia: Trace elements in men and lactating women, predictors of exposure and reference values. *Int J Hyg Environ Health*. **2019**, 222(3):563-582. https://doi:10.1016/j.ijheh.2019.02.008

11. Trdin A, Falnoga I, Fajon V, et al. Mercury speciation in meconium and associated factors. *Environ Res.* **2019**, 179(Pt A):108724. https://doi:10.1016/j.envres.2019.108724

12. Batista J, Schuhmacher M, Domingo JL, Corbella J. Mercury in hair for a child population from Tarragona Province, Spain. *Sci Total Environ.* **1996**, 193(2):143-148. https://doi:10.1016/s0048-9697(96)05340-5

13. Castaño A, Pedraza-Díaz S, Cañas AI, et al. Mercury levels in blood, urine and hair in a nation-wide sample of Spanish adults. *Sci Total Environ.* **2019**, 670:262-270. https://doi:10.1016/j.scitotenv.2019.03.174

14. Llop S, Murcia M, Amorós R, et al. Postnatal exposure to mercury and neuropsychological development among preschooler children. *Eur J Epidemiol*. **2020**, 35(3):259-271. https:// doi:10.1007/s10654-020-00620-9

15. Pérez R, Suelves T, Molina Y, Corpas-Burgos F, Yusà V; BIOVAL task force. Biomonitoring of mercury in hair of children living in the Valencian Region (Spain). Exposure and risk assessment. *Chemosphere*. **2019**, 217:558-566. https://doi:10.1016/j.chemosphere.2018.11.017

16. Yusà V, Pérez R, Suelves T, et al. Biomonitoring of mercury in hair of breastfeeding mothers living in the Valencian Region (Spain). Levels and predictors of exposure. *Chemosphere*. **2017**, 187:106-113. https://doi:10.1016/j.chemosphere.2017.08.100

17. Bocca B, Ruggieri F, Pino A, et al. Human biomonitoring to evaluate exposure to toxic and essential trace elements during pregnancy. Part B: Predictors of exposure. *Environ Res.* **2020**, 182:109108. https://doi:10.1016/j.envres.2019.109108

18. Bocca B, Ruggieri F, Pino A, et al. Human biomonitoring to evaluate exposure to toxic and essential trace elements during pregnancy. Part A. concentrations in maternal blood, urine and cord blood. *Environ Res.* **2019**, 177:108599. https://doi:10.1016/j.envres.2019.108599

19. Almerud P, Zamaratskaia G, Lindroos AK, et al. Cadmium, total mercury, and lead in blood and associations with diet, sociodemographic factors, and smoking in Swedish adolescents. *Environ Res.* **2021**, 197:110991. https://doi:10.1016/j.envres.2021.110991
